# Macrophage-derived insulin-like growth factor-1 is a key neurotrophic and nerve-sensitizing factor in pain associated with endometriosis

**DOI:** 10.1096/fj.201900797R

**Published:** 2019-07-10

**Authors:** Rachel Forster, Alexandra Sarginson, Atanaska Velichkova, Chloe Hogg, Ashley Dorning, Andrew W. Horne, Philippa T. K. Saunders, Erin Greaves

**Affiliations:** * Medical Research Council (MRC) Centre for Reproductive Health, The Queen’s Medical Research Institute, The University of Edinburgh, Edinburgh, United Kingdom;; † MRC Centre for Inflammation Research, The Queen’s Medical Research Institute, The University of Edinburgh, Edinburgh, United Kingdom;; ‡ Division of Biomedical Sciences, Warwick Medical School, University of Warwick, Coventry, United Kingdom

**Keywords:** hyperalgesia, leukocytes, neurotrophin, nerve

## Abstract

Endometriosis is a common incurable inflammatory disorder that is associated with debilitating pelvic pain in women. Macrophages are central to the pathophysiology of endometriosis: they dictate the growth and vascularization of endometriosis lesions and more recently have been shown to promote lesion innervation. The aim of this study was to determine the mechanistic role of macrophages in producing pain associated with endometriosis. Herein, we show that macrophage depletion in a mouse model of endometriosis can reverse abnormal changes in pain behavior. We identified that disease-modified macrophages exhibit increased expression of IGF-1 in an *in vitro* model of endometriosis-associated macrophages and confirmed expression by lesion-resident macrophages in mice and women. Concentrations of IGF-1 were elevated in peritoneal fluid from women with endometriosis and positively correlate with their pain scores. Mechanistically, we demonstrate that macrophage-derived IGF-1 promotes sprouting neurogenesis and nerve sensitization *in vitro*. Finally, we show that the Igf-1 receptor inhibitor linsitinib reverses the pain behavior observed in mice with endometriosis. Our data support a role for macrophage-derived IGF-1 as a key neurotrophic and sensitizing factor in endometriosis, and we propose that therapies that modify macrophage phenotype may be attractive therapeutic options for the treatment of women with endometriosis-associated pain.—Forster, R., Sarginson, A., Velichkova, A., Hogg, C., Dorning, A., Horne, A. W., Saunders, P. T. K., Greaves, E. Macrophage-derived insulin-like growth factor-1 is a key neurotrophic and nerve-sensitizing factor in pain associated with endometriosis.

Endometriosis is a chronic incurable estrogen-dependent inflammatory disorder affecting an estimated 176 million women worldwide (1). It is associated with debilitating chronic pelvic pain and infertility and can significantly impair health-related quality of life (2–4). Endometriosis is defined by the attachment and growth of endometrial-like tissue outside the uterine cavity (endometriosis lesions), and current treatment options are limited to surgical ablation or excision of lesions or medical management to suppress ovarian hormone production. However, symptoms recur within 5 yr in 40–50% of women following surgery (5), and medical management often has unwanted side-effects and is contraceptive (6). There is an unmet clinical need for new medical treatments for women with endometriosis.

Endometriosis lesions recruit sensory nerve fibers (7, 8) that innervate the ectopic endometrial tissue and can be activated by the local neuroinflammatory milieu (9). Repeated interactions between peripheral sensory afferents (nociceptors) and cytokines and neurotrophins have been shown in other chronic pain conditions to cause nerve sensitization: an enhanced responsiveness of afferents (10), leading to a resultant increase in excitability of the nervous system and triggering pain hypersensitivity or allodynia (11, 12). In a mouse model of endometriosis (13) that recapitulates changes in sensory behavior and mirrors the range of painful manifestations observed in women with endometriosis, we have demonstrated molecular alterations along the pain axis resulting from the presence of endometriosis lesions (14).

Monocytes and macrophages in tissues are known to play active roles in pain by producing a range of pronociceptive molecules. These include cytokines, neurotrophins, and prostaglandins that can activate nerves by binding to their cognate receptors, triggering intracellular signaling cascades that induce sensitization by activation or up-regulation of nociceptive ion channels such as transient receptor potential cation channel (TRP) A1, TRPV1, and the sodium ion channels Nav1.7–1.9 (10, 15, 16). The extent to which signals from macrophages generate changes in sensory behavior is dependent on the cause of pain; mechanical hypersensitivity caused by sterile incision as a model of tissue injury based inflammation is rescued by macrophage depletion, whereas monocytes seemingly have no effect (17). However, in a model of chemotherapy-induced neuropathic pain, monocytes migrate into peripheral nerves and produce reactive oxygen species that generate pain by activating TRPA1 (18). In disease models of pain, macrophages play a key role. For example, in an osteoarthritis model, C-C motif chemokine receptor 2 (Ccr2) signaling, a key driver of macrophage recruitment, is required for movement-provoked pain behaviors (19), and in mice prone to lupus (systemic lupus erythematosus), blocking macrophage colony stimulating factor (m-csf; a factor critical for macrophage recruitment and survival) can attenuate thermal hyperalgesia (20). In rats with diabetic neuropathy, macrophages have also been implicated in eliciting a pain response; depletion of macrophages after traumatic or metabolic nerve injury significantly reduces or prevents the progression of mechanical hyperalgesia and allodynia (21).

Macrophages are considered central players in the pathophysiology of endometriosis, dictating both proliferation and vascularization of lesions (22, 23). They are observed clustered around nerve fibers in endometriosis lesions (24), and we have demonstrated a functional 2-way interaction between macrophages and nerves in endometriosis that is mediated by E_2_ (25), a ligand that is generated in lesions by overexpression of steroidogenic enzymes, including aromatase (26). Specifically, an E_2_-dependent increase in chemokine ligand 2 (Ccl-2) by nerve fibers recruits macrophages, which exhibit an increase in expression of brain-derived neurotrophic factor (Bdnf) and neurotrophin-3 (Nt-3), leading to concomitant neurotrophic effects on nerves (25). Although macrophages may promote nerve growth in endometriosis lesions, it is not known if they contribute to endometriosis-associated pain. Thus, we hypothesized that macrophages contribute to endometriosis-associated pain by secreting factors that encourage nerve growth and sensitization.

## MATERIALS AND METHODS

### Animals and reagents

C57BL/6 mice were purchased from Harlan (Indianapolis, IN, USA). To achieve macrophage depletion, liposomal clodronate (Encapsula NanoSciences, Brentwood, TN, USA) or saline (controls) were injected intraperitoneally in 100 μl volume. Liposomes were administered every 48 h. Linsitinib, an IGF-1 receptor (IGF-1R) inhibitor with modest activity of the insulin receptor (40 mg/kg; Selleckchem, Houston, TX, USA), or vehicle (30% polyethylene glycol 400, 0.5% Tween-80, and 5% propylene glycol) was administered by oral gavage every 24 h. Mice had access to food and water *ad libitum*. Ambient temperature and humidity were 21°C and 50%, respectively.

### Mouse model of endometriosis

**Figure 1:**
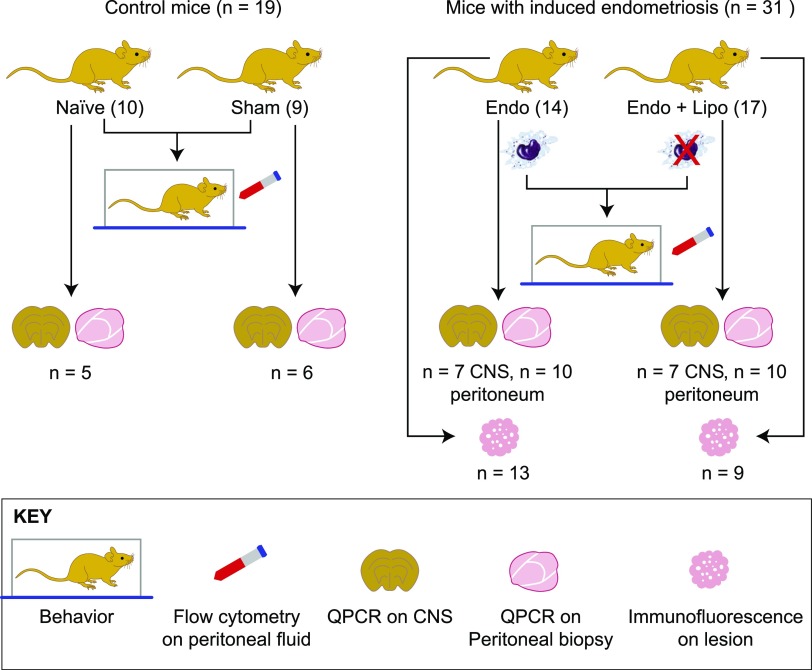
Flow diagram of mice used for each experimental endpoint. Control mice [*n* = 19; naive (*n* = 10); sham-treated mice (*n* = 9)] and mice with induced endometriosis [*n* = 31; Endo (*n* = 14), Endo + Lipo (*n* = 17)] were used for behavior assessments and flow cytometry analysis of macrophage populations. Of these, spinal cord, brain, and peritoneal biopsies were collected from *n* = 5 naive and *n* = 6 sham-treated mice. Spinal cord and brain were collected from *n* = 7, and peritoneal biopsies were collected from *n* = 10 Endo and Endo + Lipo mice. CNS and peritoneal tissue were subject to gene expression analysis using qPCR. We recovered lesions from 13 (out of 14) Endo mice and 9 (out of 17) Endo + Lipo mice, and these were used for immunofluorescence. CNS, central nervous system (spinal cord and brain); Lipo, liposomes.

Endometriosis was induced in mice as previously described by Greaves *et al*. (13). In brief, donor mice were induced to undergo endometrial breakdown in a menses-like event (27). Donor mice were culled, and the endometrial tissue was collected by opening the decidualized uterine horn and scraping the endometrium away from the underlying myometrium. Approximately 40 mg of endometrial tissue was injected into the peritoneal cavity of E_2_-primed (500 ng E_2_ valerate) recipient mice. Experiment 1 (macrophage depletion): 21 d post endometrial tissue injection, mice with induced endometriosis (*n* = 31) were randomly assigned to 1 of 2 groups: liposomal clodronate (*n* = 17) or saline (*n* = 14). Behavior assessments were performed to ascertain pretreatment recordings. Macrophage depletion was started on d 21 of the endometriosis protocol and maintained for an additional 7 d (21–28 d). At 28 d, mice were culled, and the following samples were recovered (see **Fig. 1** for flow diagram accounting for sample size for each endpoint): peritoneal lavage (*n* = 14 and 17; recovered by injecting 7 ml ice-cold DMEM into the peritoneal cavity followed by gentle massage and recovery), peritoneal biopsy (*n* = 10 each group), endometriosis lesions (*n* = 13 and 9), lumbar spinal cord (T13-L5; *n* = 7), and medial prefrontal cortex (brain; *n* = 7). Samples were also collected from naive (*n* = 10) and sham-treated (ovariectomy + E_2_ + intraperitoneal injection of PBS; *n* = 9) animals. Samples were collected into RNAlater (Thermo Fisher Scientific, Waltham, MA, USA) and frozen for quantitative PCR (qPCR) analysis (peritoneum, spinal cord, and brain), neutral-buffered formalin for paraffin embedding, and immunohistochemical analysis (endometriosis lesions). Suspected lesions were stained using hematoxylin and eosin and assessed for the presence of stroma^+/−^ glands. Biopsies that did not include either epithelial or stromal compartments were not included in any further analysis. Experiment 2 (Igf-1r inhibition): In a separate experiment, mice with induced endometriosis (*n* = 24) were randomly assigned to 1 of 2 groups (vehicle, *n* = 12 or linsitinib, *n* = 12), and behavior assessments were performed.

### Behavior assessments

Behavior assessments were performed as we have previously described in detail (14). In this study, behavioral assessments were performed in a blinded fashion, and all animals were acclimatized to the apparatus and handling prior to the initiation of behavior analysis. Spontaneous (grooming and activity) and evoked (mechanical hyperalgesia measured using von Frey filaments) behaviors were recorded. Experiment 1: Assessments were performed on 31 mice with induced endometriosis (saline, *n* = 14; liposomal, *n* = 17). Groups of naive (*n* = 10) and sham-treated (ovariectomy + E_2_ + intraperitoneal injection of PBS; *n* = 9) animals were included in all assessments. Experiment 2: Assessments were performed on 24 mice with endometriosis (vehicle, *n* = 12; linsitinib, *n* = 12), naive (*n* = 12), and sham-treated (*n* = 6) mice.

### Patients and recovery of tissue and fluid samples

Endometriosis lesions (*n* = 8; proliferative phase, *n* = 4; and secretory phase, *n* = 4) and peritoneal fluid (PF) (*n* = 13; proliferative phase, *n* = 6; secretory phase, *n* = 7) were collected from women undergoing laparoscopic investigation for chronic pelvic pain with presence of endometriosis confirmed at the time of surgery. PF was also collected from women undergoing investigation for chronic pelvic pain with the absence of endometriosis (*n* = 9; proliferative phase, *n* = 5; secretory phase, *n* = 4). None of the women had taken exogenous hormones for ≥3 mo at the time of sampling. Samples were collected, stored, and processed in accordance with Endometriosis Phenome and Biobanking Harmonisation Project (EPHect) guidelines (28–31). Endometriosis lesions were fixed in 4% neutral-buffered formalin and paraffin embedded. PF samples were centrifuged to pellet any cells and debris, divided into aliquots, and frozen at −80°. Pain scores were calculated using the Endometriosis Health Profile 30 questionnaire.

### *In vitro* generation of endometriosis-associated macrophages

To isolate mononuclear leukocytes, blood was processed using dextran sedimentation and separated on a Percoll (GE Healthcare, Waukesha, WI, USA) gradient with negative selection as previously described by Greaves *et al*. (25). Adherent monocytes were cultured on tissue culture plates in the presence of 4 ng/ml M-CSF 1 for 7 d to generate monocyte-derived macrophages (MDMs). Cells were cultured in DMEM (Thermo Fisher Scientific) containing 10% AB human serum (AMS Biotechnology, Milton, United Kingdom) in 12-well plates maintained at 37°C in 5% CO_2._ After differentiation to macrophages, MDMs were activated with PF from patients with (*n* = 7; secretory phase) or without (*n* = 8; secretory phase) endometriosis diluted 1:1 in medium for 24 h. Macrophages activated with PF from patients with endometriosis are referred to as *in vitro* generated endometriosis-associated macrophages (EAMs). We also included prototypically polarized macrophages as controls; from each blood preparation, some macrophages were activated with 20 ng/ml IFN-γ and 50 ng/ml LPS to generate proinflammatory macrophages and others were activated with 20 ng/ml IL-4, IL-10, and TGF-β to generate prorepair macrophages; unactivated macrophages (M0s; medium only) were also included as controls. Conditioned medium was collected by removing PF or medium containing cytokines, washing once in fresh medium, and then incubating in medium for an additional 24 h. The conditioned medium was collected and frozen in aliquots at Oct4-80°C.

### Human embryonic stem cell differentiation to sensory neurons

Human embryonic stem cells (hESCs), strain H9 (WiCell, Madison, WI, USA) were maintained and differentiated into sensory neurons using small molecule inhibitors as previously described in refs. 32 and 33. Differentiation was verified by confirming down-regulation of the pluripotency marker octamer-binding transcription factor 4 and up-regulation of the nociceptive genes tachykinin precursor 1 (TAC1), sodium voltage-gated channel (SCN) 9A, and SCN11A. Functionality of sensory neurons was confirmed by stimulating cells with 4 nM capsaiscin (MilliporeSigma, Burlington, MA, USA) and recording intracellular calcium flux using a calcium indicator kit (BD Biosciences, San Jose, CA, USA), with calcium flux captured using a Novostar microplate fluorometer (BMG Labtech, Cary, NC, USA).

### Neuronal outgrowth assay

Embryonic rat dorsal root ganglia (DRGs) were isolated as previously described by Greaves *et al*. (34). Whole-ganglion explants were plated onto poly-D-lysine- and Matrigel-coated wells (BD Biosciences) in 48-well plates and incubated in DRG medium (high-glucose DMEM supplemented with 0.01% penicillin-streptomycin, 10% fetal calf serum). Positive controls were supplemented with nerve growth factor (NGF) (Bio-Rad, Hercules, CA, USA) plus recombinant IGF-1 (R&D Systems, Minneapolis, MN, USA) (2–200 ng/ml) ± 125–500 nM Picropodophyllin (PPP; Tocris Bioscience, Bristol, United Kingdom). Some DRGs were incubated in PF or macrophage-conditioned medium diluted 1:1 in DRG medium ± 500 nM PPP. Images of explants were captured using an Axiovert microscope (Carl Zeiss, Oberkochen, Germany), an Axiovision camera, and software.

### Flow cytometry

Red blood cells were lysed from peritoneal lavages, and equal numbers of cells were blocked with 0.025 μg anti-CD16/32 (clone 93; BioLegend, San Diego, CA, USA) and then stained with a combination of antibodies shown in **Table 1**. Fluorescence minus 1 and negative controls were used to confirm gating strategies. Just prior to analysis, DAPI and 123count eBeads allowing absolute numbers of cells to be determined (Thermo Fisher Scientific) were added to samples. Samples were acquired using an LSRFortessa with FACSDiva software (BD Biosciences) and analyzed with FlowJo v.9 software (FlowJo, Ashland, OR, USA). Analysis was performed on single live cells determined using forward scatter height *vs.* area and negativity for live or dead (DAPI).

**TABLE 1:** Flow cytometry antibodies

Antibody	Target cell	Fluorochrome	Supplier	Dilution (v/v)
CD3	T cells	APC	BioLegend	1:100
B220	B cells	APC	BioLegend	1:100
NKp46	NK cells	APC	BioLegend	1:100
Siglec-F	Eosinophils	APC	BD Biosciences	1:100
Cd11b	Granulocytes	BV650	BioLegend	1:100
CD45	Leukocytes	PE/Dazzle594	BioLegend	1:1000
Ly6C	Monocytes	Pacific Blue	BioLegend	1:100
Ly6G	Neutropils	PE/Cy7	BioLegend	1:50
Cd11c	Dendritic cells	PERCP/Cy5.5	BioLegend	1:100
F4/80	Macrophages	Alexa Fluor 488	BioLegend	1:50
DAPI				1:40,000

APC, allophycocyanin; BV650, Brilliant Violet 650; Ly6G, lymphocyte antigen 6 complex, locus G; PE, phycoerythrin; PERCP, peridinin chlorophyll protein.

### Real-time qPCR

RNA was extracted from human and mouse tissues by homogenization in Trizol reagent and chloroform phase separation prior to processing using an RNAeasy Kit (Qiagen, Hilden, Germany). RNA was extracted from cells using RLT (lysis) buffer and an RNAeasy Kit. Concentration and purity were assessed using a Nanodrop 1000 (Thermo Fisher Scientific). A standard curve was generated by pooling undiluted RNA samples and performing four 10-fold dilutions. cDNA was synthesized using SuperScript Vilo Enzyme (Thermo Fisher Scientific) with 100 ng starting template in a 20-μl reaction. PCRs (10 μl) were performed using the Roche Universal Library (Roche, Basel, Switzerland) and Express qPCR Supermix (Thermo Fisher Scientific). cDNA was added at 1 μl per reaction, forward and reverse primers (**Table 2**) were added at 20 μM, and thermal cycling conditions were performed on a 7900 Fast real-time PCR machine (Thermo Fisher Scientific) in 384-well plates with technical duplicates performed. 18S (Thermo Fisher Scientific) was selected as the reference gene. Data were analyzed using the relative standard curve method, and samples were normalized to 1 consistent sample.

**TABLE 2:** Primer sequences

	Primer sequence, 5′–3′		
Gene	Forward	Reverse	UPL probe
*Cox-2*	GATGCTCTTCCGAGCTGTG	GGATTGGAACAGCAAGGATTT	45
*Tnf-α*	CTGTAGCCCACGTCGTAGC	TTTGAGATCCATGCCGTTG	25
*Igf-1*	AGCAGCCTTCCAACTCAATTAT	GAAGACGACATGATGTGTATCTTTATC	34
*BDNF*	GTAACGGCGGCAGACAAA	GACCTTTTCAAGGACTGTGACC	86
*NT-3*	CCCTTGTATCTCATGGAGGATT	TTTCCGCCGTGATGTTCT	44
*IGF-1*	TGTGGAGACAGGGGCTTTTA	ATCCACGATGCCTGTCTGA	67
*SCN9A*	CAACTTTTAAGGGATGGACGA	TCATATTTGGGCTGCTTGTCT	86
*SCN11A*	ACCTGAGCCTGAACAACAGG	TTTGAACTCTCTGGCTCGTG	2
*TAC1*	GCCTCAGCAGTTCTTTGGAT	AGCCTTTAACAGGGCCACTT	89

Cox-2, cyclooxygenase-2; UPL, Universal Probe Library.

### Immunofluorescence

Sections were antigen retrieved using citrate buffer, heat, and pressure (pH 6.0 for CD68 or pH 9.0 for IGF-1) or trypsin tablets dissolved in dH_2_O (for F4/80; MilliporeSigma) incubated with sections for 20 min at 37°C. Sections were blocked for endogenous peroxidase and nonspecific epitopes (species-specific serum diluted 1:5 in Tris-buffered saline and 5% bovine serum albumin) and incubated with primary antibody (**Table 3**) at 4°C overnight. Antibody detection was performed using a secondary pAb to IgG (horseradish peroxidase) and a tyramide signal amplification system kit with cyanine (Cy) 3 or fluorescein (1:50 dilution; PerkinElmer, Waltham, MA, USA). For detection of the second antigen in dual immunofluorescence, sections were boiled in citrate buffer, and the second primary antibody was applied overnight and detected as before. Prior to mounting in Permafluor (Thermo Fisher Scientific), sections were counterstained with DAPI. Images were captured using an LSM710 confocal microscope and AxioCam camera (Carl Zeiss). Human or mouse uterus was used as a positive control tissue, and negative controls had omission of the primary antibody.

**TABLE 3:** Primary antibodies used in immunofluorescence

Antibody	Host	Species	Supplier	Dilution (v/v)
F4/80	Rat	Mouse	Thermo Fisher Scientific	1:600
CD68	Mouse	Human	Dako	1:800
IGF-1	Rabbit	Human	Santa Cruz Biotechnology	1:150
Neurofilament	Chicken	Rat	Covance	1:1000

### IGF-1 ELISA

IGF-1 levels were detected in conditioned medium and PF using a Human IGF-1 DuoSet ELISA (R&D Systems) according to the manufacturer’s instructions.

### Statistics

Initially, data were tested for normality using Shapiro-Wilk and Kolmogorov-Smirnov tests. Statistical analysis was performed using a Student’s *t* test or Mann-Whitney *U* test (nonnormal data) to compare 2 experimental groups or a 1-way ANOVA with a Tukey’s multiple comparison test to compare ≥3 experimental groups. For von Frey data, medians were plotted, and a Kruskal-Wallis test with a Dunn’s multiple comparison test was performed.

### Study approval

Mouse experiments were permitted under license by the United Kingdom Home Office and were approved by the University of Edinburgh Animal Welfare and Ethical Review Body (Edinburgh, United Kingdom). Behavior assessments were performed in accordance with the Guidelines of the Committee for Research and Ethical Issues of the International Association for the Study of Pain. For the collection of patient biopsies, the study was approved by the Lothian Research Ethics Committee (LREC 11/AL/0376), and all samples were collected after informed consent was obtained in accordance with EPHect guidelines (29, 30). Human venous blood was collected from healthy female volunteers (*n* = 7) with informed consent and approval from the Local Lothian Research Ethics Committee (AMREC 15-HV-013).

## RESULTS

### Macrophages play a key role in endometriosis-associated hyperalgesia in mice with induced endometriosis

**Figure 2:**
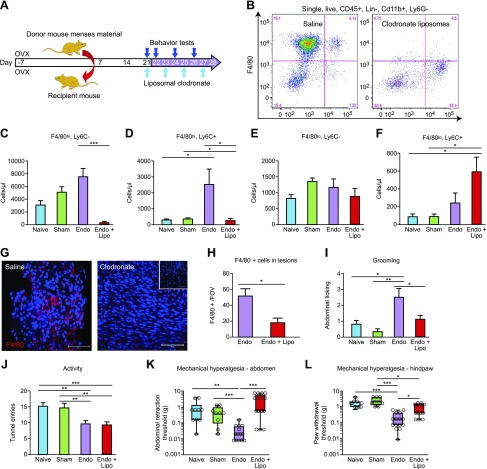
Macrophages play a key role in endometriosis-associated hyperalgesia. *A*) Schematic of endometriosis model indicating timing of behavioral tests and liposome–saline administration. *B*) Representative flow plots showing peritoneal lavage of saline control (left panel) and liposomal clodronate depleted (right panel). Myeloid cells were separated into 4 populations using F4/80 and Ly6C; these were F4/80^hi^, Ly6C^−^ (LpMs), F4/80^hi^, Ly6C^+^ (MDMs), F4/80^lo^, Ly6C^lo^ (SpMs), and Ly6C^+^, F4/80^−^ (monocytes**).** *C***–***F*) Quantification of flow cytometry results normalized to 123count eBeads to allow resolution of absolute numbers (naive mice, *n* = 10; sham-treated mice, *n* = 9; Endo, *n* = 14; Endo + Lipo, *n* = 17). *C*, *D*) Clodronate administration caused depletion of LpMs (*P* < 0.001) (*C*) and MDMs (*P* < 0.05) (*D*). *E*) SpMs were not significantly altered. *F*) Monocytes were significantly increased in depleted endometriosis mice compared with naive and sham-treated animals (*P* < 0.05). *G*) Macrophages in the lesions of mice were visualized using F4/80 (red) immunofluorescence. Scale bar, 50 μM. Inset on right panel is negative control (negative primary antibody). *H*) Macrophage numbers [3 × random fields of view (FOVs) per lesion quantified] were reduced (*P* < 0.01) in the lesions of mice that received liposomal clodronate (*n* = 9 mice) compared with saline (*n* = 13 mice). *I*) In C57BL/6 mice with endometriosis (*n* = 14), abdominally directed grooming was elevated compared with naive (*n* = 10; *P* < 0.05) and sham-treated animals (*n* = 9; *P* < 0.01). Macrophage depletion (*n* = 17) decreased grooming levels (*P* < 0.05). *J*) Exploratory behavior was reduced in mice with endometriosis (*P* < 0.01). Macrophage depletion did not rescue endometriosis-associated changes in activity. *K*) Macrophage depletion increased abdominal (*P* < 0.01) retraction thresholds. *L*) Paw withdrawal thresholds were lower in endometriosis mice compared with naive and sham-treated animals (*P* < 0.001), and macrophage depletion attenuated hind-paw withdrawal thresholds. A single time point (d 26) that exhibited maximal changes in sensory behavior is shown. A Mann-Whitney *U* test, (*H*), 1-way ANOVA, and Tukey’s multiple comparison test (*I*, *J*) or a Kruskal-Wallis nonparametric test with Dunn’s multiple comparison test were performed (*K*, *L*). Lin, lineage; Lipo, liposome; OVX, ovariectomy. *P < 0.05, **P < 0.01, ***P < 0.001.

In C57BL/6 mice with induced endometriosis, macrophages were depleted on d 21 post tissue injection using liposomal clodronate. Injections were repeated every 48 h (d 23, 25, and 27) to maintain depletion, and the mice were culled on d 28 (**Fig. 2*A***). To confirm depletion of macrophages, we performed flow cytometry on cells isolated from peritoneal lavage. Among CD45^+^, CD3^−^, CD19^−^, NKp46^−^, Siglec-F^−^, lymphocyte antigen 6 complex, locus G^−^ (Ly6G), and Cd11b^+^ cells, 4 populations were separated based on expression of F4/80 and lymphocyte antigen 6 complex, locus C (Ly6C) (Fig. 2*B*). These were large peritoneal macrophages (LpMs; F4/80^hi^, Ly6C^−^), a population of F4/80^hi^ Ly6C^+^ cells that likely represent a subset of transient MDMs, and small peritoneal macrophages (SpMs; F4/80^lo^, Ly6C^−^) and monocytes (F4/80^−^, Ly6C^+^). Administration of liposomal clodronate induced a significant depletion of LpMs (Fig. 2*C*; *P* < 0.001) and transient MDMs (Fig. 2*D*; *P* < 0.05). There was no significant difference in numbers of SpMs (Fig. 2*E*). Lipsomal depletion of macrophages in mice with endometriosis induced a significant increase in the number of monocytes compared with naive and sham-treated mice (Fig. 2*F*), but monocyte numbers were not significantly different to (nondepleted) mice with endometriosis. We observed a significant increase in the number of LpMs in mice with endometriosis compared with naive animals (Fig. 2*C*; *P* < 0.01). We recovered lesions from 13 (out of 14) mice treated with saline and 9 (out of 17) mice treated with liposomal clodronate. Using immunofluorescence, we identified a reduction in F4/80^+^ macrophages present in endometriosis lesions compared with control animals (Fig. 2*G, H*; *P* < 0.05). Mice with endometriosis exhibited increased levels of spontaneous abdominal grooming (Fig. 2*I*; *P* < 0.01) and decreased levels of activity (Fig. 2*J*; *P* < 0.01) as well as decreased abdominal retraction (Fig. 2*K*; *P* < 0.001) and paw withdrawal thresholds (Fig. 2*L*; *P* < 0.001) when stimulated with a punctate stimulus (von Frey filaments; an evoked measure of mechanical hyperalgesia) compared with naive or sham-treated animals [consistent with what we have observed previously (14)]. Following macrophage depletion with liposomal clodronate, the levels of grooming in the endometriosis mice declined such that they were no longer different to those observed in naive and sham-treated animals (Fig. 2*I*; *P* < 0.05 compared with mice with induced endometriosis). Macrophage depletion did not rescue activity levels in endometriosis mice (Fig. 2*J*). However, there was a significant difference in abdominal retraction threshold between nondepleted and macrophage-depleted endometriosis mice (Fig. 2*K*; *P* < 0.001), with endometriosis mice withdrawing from a lighter stimulus than depleted animals. Depletion of macrophages also attenuated paw withdrawal thresholds in endometriosis mice (Fig. 2*L*; *P* < 0.05 compared with nondepleted endometriosis mice). Thus, it appears that macrophages play a key role in altered sensory behavior in mice with endometriosis.

### Depletion of macrophages attenuates markers of inflammatory pain hypersensitivity in the CNS of mice with induced endometriosis

**Figure 3:**
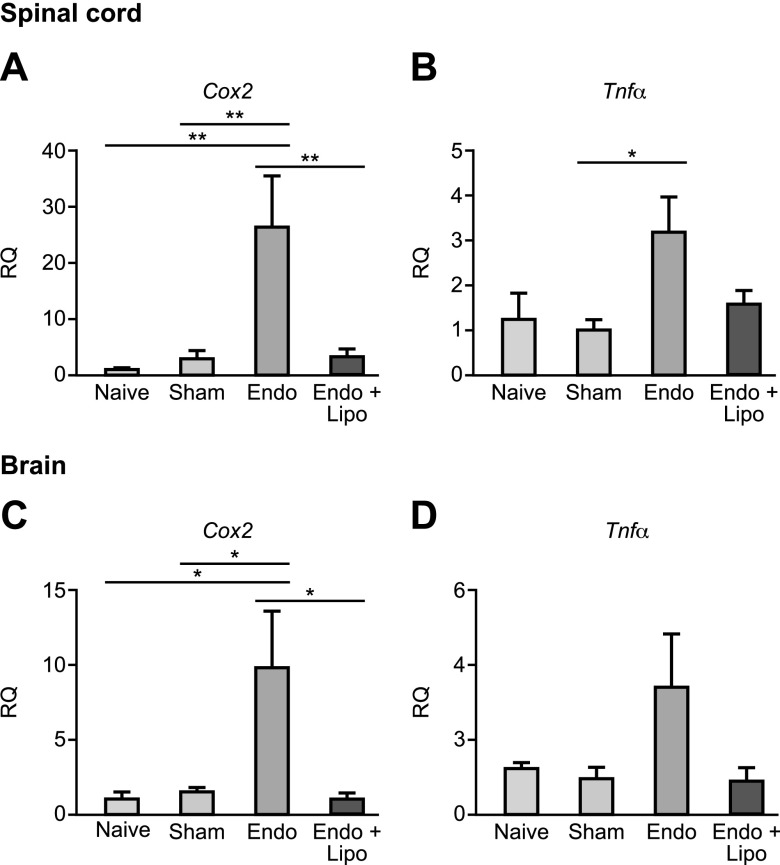
Peripheral macrophages mediate endometriosis-associated inflammation in the CNS. qPCR analysis revealed evidence of apparent changes in the mRNA concentrations of key inflammatory genes in the spinal (T13-L5 segments) and medial prefrontal cortex area of the brains of mice with endometriosis. *A*) In the spinal cords of mice with endometriosis (*n* = 7), *Cox-2* mRNA was significantly elevated (*P* < 0.01) compared with naive (*n* = 5) and sham-treated (*n* = 6) mice controls. This was attenuated following macrophage depletion (*n* = 7; *P* < 0.01). *B*) *Tnf-α* mRNA concentrations were increased in the spinal cords of mice with endometriosis (*P* < 0.05), and macrophage depletion attenuated levels. *C*) *Cox-2* was also elevated in the brain (medial prefrontal cortex; *P* < 0.05) of mice with endometriosis, and macrophage depletion reduced expression (*P* < 0.05). *D*) *Tnf-α* was increased but not significantly in the brain, and macrophage depletion reduced expression levels. Cox-2, cyclooxygenase-2; Lipo, liposomes; RQ, relative quantification.

We have previously shown that the presence of endometriosis lesions leads to increased expression of nociceptive and inflammatory markers (Trpv1, Scn11A, and Cox-2) in DRGs and spinal cords and the brains of mice with endometriosis (14). Cox-2 has previously been implicated as a marker of inflammatory pain hypersensitivity (35). In line with our previous findings, endometriosis mice exhibited increased mRNA expression of *Cox-2* (**Fig. 3*A***; *P* < 0.01) as well as *Tnf-α* (Fig. 3*B*; *P* < 0.05) in the spinal cord compared with naive and sham-treated animals, and these levels were attenuated following macrophage depletion using liposomal clodronate. The medial prefrontal cortices of the brains of mice with endometriosis also exhibited apparent increased mRNA expression of inflammatory genes, with *Cox-2* being significantly different to controls (Fig. 3*C*; *P* < 0.05). Expression of *Cox-2* was reversed by macrophage depletion (*P* < 0.05). *Tnf-α* levels in the brain were not significantly altered following macrophage depletion (Fig. 3*D*). We conclude that detectable molecular markers of inflammatory pain in the nervous system of mice with endometriosis can be attenuated by macrophage depletion.

### Disease-modified macrophages in endometriosis exhibit elevated expression of IGF-1

**Figure 4:**
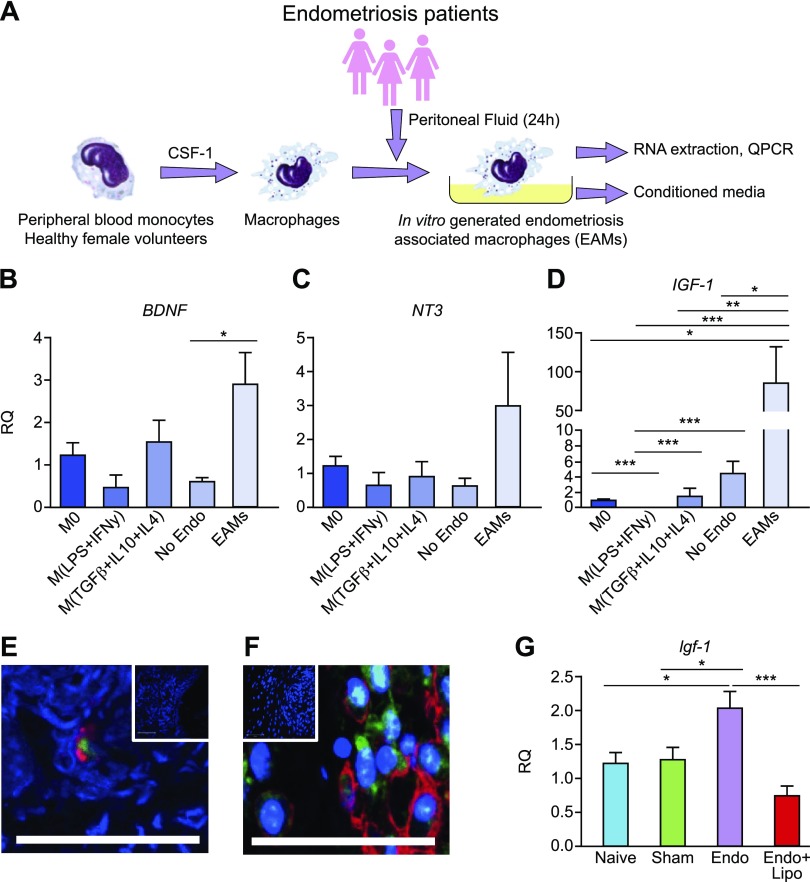
Disease-modified macrophages in endometriosis exhibit elevated expression of IGF-1. *A*) Schematic showing generation of EAMs: peripheral blood monocytes from healthy female volunteers (*n* = 7) were differentiated into macrophages for 7 d in the presence of recombinant M-CSF 1. Macrophages were then activated for 24 h with PF from patients with endometriosis (*n* = 7) or without endometriosis (*n* = 8) or activated with different cytokines to generate inflammatory (LPS + IFN-γ) or repair (TGF-β + IL-10 + IL-4; all *n* = 7) macrophages and compared with M0s. *B–D*) qPCR revealed that EAMs had significantly increased mRNA concentration of *BDNF* (*P* < 0.05) (*B*), elevated levels of *NT-3* (*C*), and significantly increased concentrations of *IGF-1* (*P* < 0.001) (*D*). *E*) Dual immunofluorescence for CD68 (macrophages; red) and IGF-1 (green) revealed that macrophages in human endometriosis lesions express IGF-1. *F*) In lesions recovered from mice with induced endometriosis, we identified F4/80^+^ macrophages (red) that stained positive for Igf-1 (green). Scale bar, 50 μM. Top insets show negative controls where the primary antibody is omitted. *G*) In peritoneal biopsies recovered from endometriosis mice (*n* = 10), qPCR analysis revealed that mRNA concentration of *Igf-1* was elevated (*P* < 0.05) compared with naive (*n* = 5) and sham-treated (*n* = 6) animals. Macrophage depletion in endometriosis mice (*n* = 10) significantly reduced *Igf-1* expression (*P* < 0.001). RQ, relative quantification. *P < 0.05, **P < 0.01, ***P < 0.001.

To model EAMs *in vitro*, we activated human peripheral blood MDMs (see Supplemental Fig. S1 for characterization of monocyte-macrophage differentiation) collected from healthy female volunteers with PF from patients with endometriosis (**Fig. 4*A***). M0s, proinflammatory macrophages [M(LPS+IFN-γ)], prorepair macrophages [M(TGF-β + IL-10 + IL-4)], and macrophages activated with PF from women without endometriosis [M(No Endo)] were included for comparison. In order to investigate factors produced by macrophages that may contribute to pain in endometriosis, we analyzed mRNA expression of key neurotrophic genes. mRNA expression of *BDNF* was elevated in EAMs compared with M(No Endo) (Fig. 4*B*; *P* < 0.05). Concentrations of *NT-3* also exhibit elevated levels in EAMs but this data did not reach statistical significance (Fig. 4*C*). mRNA concentrations of *IGF-1* were significantly elevated in EAMs (*P* < 0.001) compared with all other macrophages, including macrophages activated with PF from women without endometriosis [M(No Endo); *P* < 0.05, Fig. 4*D*)]. We aimed to further validate these data using patient biopsies. In endometriosis lesions recovered at surgery from women during the secretory (progesterone-dominated) phase, we could detect macrophages (CD68) that coexpressed IGF-1 using dual immunofluorescence (Fig. 4*E*). We also confirmed expression of Igf-1 in F4/80^+^ macrophages in mouse endometriosis lesions using immunofluorescence (Fig. 4*F*). In support of these findings, we also demonstrated that *Igf-1* mRNA concentrations were elevated in peritoneal biopsies of mice with endometriosis, and levels were significantly attenuated when macrophages were depleted (Fig. 4*G*; *P* < 0.001).

### IGF-1 is elevated in the PF of women with endometriosis and correlates with their pain scores

**Figure 5:**
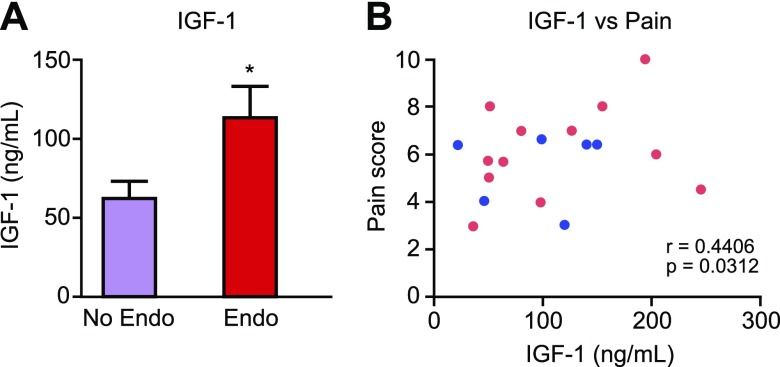
IGF-1 is elevated in the PF of women with endometriosis, and concentrations positively correlate with pain score. *A*) ELISA analysis of PF from patients with endometriosis (*n* = 13; ENDO) revealed an increase (*P* < 0.05) in protein concentrations of IGF-1 compared with patients without endometriosis (*n* = 9; No Endo). *B*) We detected a positive correlation between patient self-reported pain score [in women with chronic pelvic pain and endometriosis (pink dots) and chronic pelvic with no obvious pathology (blue dots)] and IGF-1 protein concentrations. Statistical analysis was performed using a Student’s *t* test or Pearson’s *r* test for correlation. *P < 0.05.

We aimed to further explore the importance of IGF-1 in the mechanism behind endometriosis-associated pain in women using human samples of PF. IGF-1 protein concentration was significantly elevated in the PF of women with endometriosis compared with those without disease (**Fig. 5*A***; *P* < 0.05) regardless of cycle phase (Supplemental Fig. S2*A*). We also found that concentrations of IGF-1 in PF positively correlated with patient-reported pain scores (collected prior to surgery) in women with pelvic pain but no endometriosis and women with endometriosis and pelvic pain (Fig. 5*B*; *P* < 0.05). Thus, we hypothesized that macrophage-derived IGF-1 might be a key factor involved in producing pain in endometriosis.

### Macrophage-derived IGF-1 enhances sprouting neurogenesis and nociceptive gene expression *in vitro*

**Figure 6:**
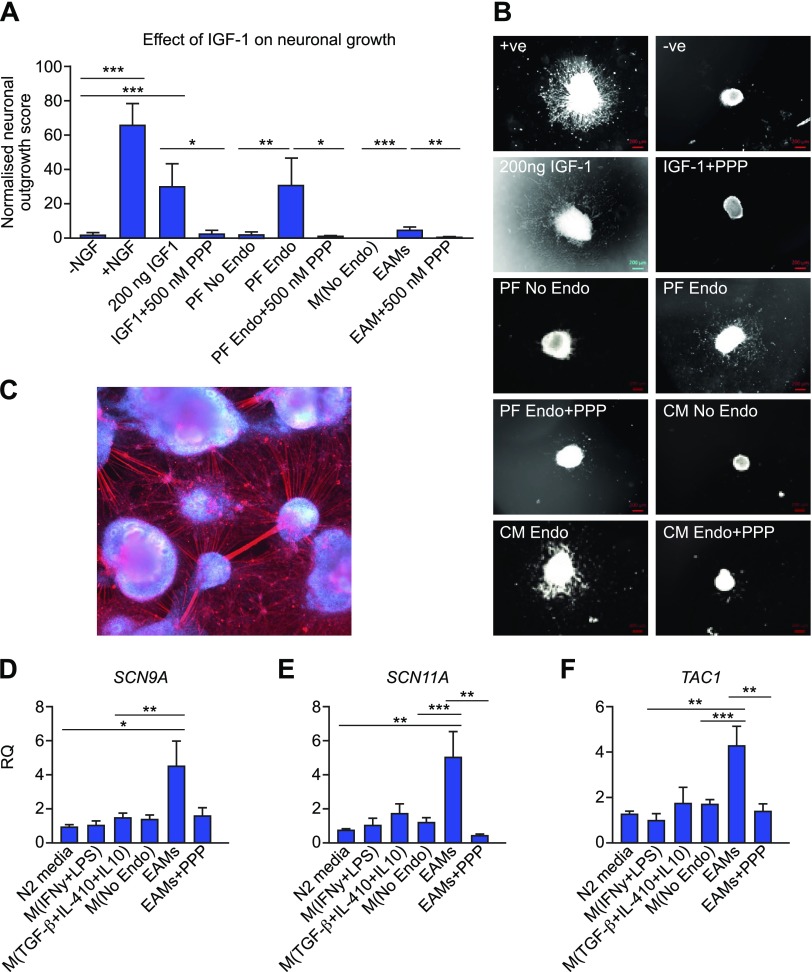
Macrophage-derived IGF-1 enhances neuronal outgrowth and nociceptive gene expression *in vitro*. *A*) Quantification of neuronal outgrowth from rat DRG explants (between 18 and 40 explants per group from embryos derived from 5 pregnant dams). Outgrowth was maximal in the presence of recombinant NGF (positive control; *P* < 0.001 compared with negative control, which was DRG medium in the absence of NGF). Recombinant IGF-1 (200 ng) also supported neuronal outgrowth (*P* < 0.05 compared with negative control), and this was abrogated by coincubation with 500 nM PPP (specific IGF-1R inhibitor; *P* < 0.05). PF from patients with endometriosis (*n* = 7) significantly enhanced neuronal outgrowth compared with PF from patients without endometriosis (*n* = 8; *P* < 0.05). This outgrowth was reduced following addition of PPP. Conditioned medium from *in vitro* generated EAMs (*n* = 7) significantly enhanced neuronal outgrowth (*P* < 0.05) compared with conditioned medium from macrophages activated with PF from patients without endometriosis [M(No Endo)]; this was reduced following addition of PPP. *B*) Representative images of whole DRG explants following incubation under different conditions. +ve, DRG medium with recombinant NGF; −ve, DRG medium without NGF; CM, conditioned medium. Scale bar, 200 μM. *C*) Human sensory neurons were differentiated from H9 hESCs using small molecular inhibitors as previously described by Chambers *et al*. (32). Axons are visualized using with neurofilament (red), and nuclei are stained with DAPI. *D*, *E*) qPCR analysis revealed that incubation of sensory neurons with EAM-conditioned medium induced an increase in mRNA concentrations of *SCN9A* (encodes Nav1.7; *P* < 0.01) (*D*) and *SCN11A* (encodes Nav1.9; *P* < 0.001) (*E*); this increase was abrogated following incubation with PPP (*P* < 0.01). *F*) mRNA concentrations of the neuropeptide *TAC1* (encodes substance P) were increased following incubation with EAM-conditioned medium (*P* < 0.001); this was reduced with PPP. *P < 0.05, **P < 0.01, ***P < 0.001.

IGF-1 is a known neurotrophic and sensitizing factor (36, 37). To determine potential mechanistic roles for macrophage-derived IGF-1 in endometriosis-associated pain, we explored the effects of EAM-conditioned medium on neuronal cell cultures. Recombinant IGF-1 (200 ng) stimulated sprouting neurogenesis in embryonic rat whole DRG explants (*P* < 0.001); this was specifically inhibited by 500 nM PPP (IGF-1R inhibitor; *P* < 0.05; **Fig. 6*A, B***). PF from women with endometriosis (PF Endo) and conditioned medium from EAMs also stimulated nerve growth compared with PF from women without disease (PF No Endo; Fig. 6*A, B*; *P* < 0.01) or conditioned medium from macrophages activated with PF from women without disease [M(No Endo); *P* < 0.001]. Sprouting neurogenesis was inhibited following addition of 500 nM PPP in each case (*P* < 0.05 and *P* < 0.01, respectively). Thus, the neurotrophic effects of PF and macrophages in endometriosis are at least in part mediated by IGF-1. hESC-derived sensory neurons (Fig. 6*C*) express ion channels that are functionally active (32, 33). Incubation with EAM-conditioned medium enhanced mRNA expression of the nociceptive sodium voltage-gated ion channels SCN9A (Fig. 6*D*; *P* < 0.01) and SCN11A (Fig. 6*E*; *P* < 0.001) but not SCN3A, the vanilloid channel TRPV, or the purinergic channel purinergic receptor P2X 3 (Supplemental Fig. S3*A–C*). The neuropeptide Substance P (encoded by the gene TAC1) was significantly up-regulated (Fig. 6*F*; *P* < 0.001), but calcitonin gene-related peptide was not (Supplemental Fig. S3*D*). Changes in gene expression were attenuated by IGF-1R inhibition *via* PPP (*P* < 0.01). Thus, we have shown a role for IGF-1 derived from EAMs in contributing to nerve growth and sensitization *in vitro*.

### Igf-1r inhibition attenuates hyperalgesia in mice with induced endometriosis

**Figure 7:**
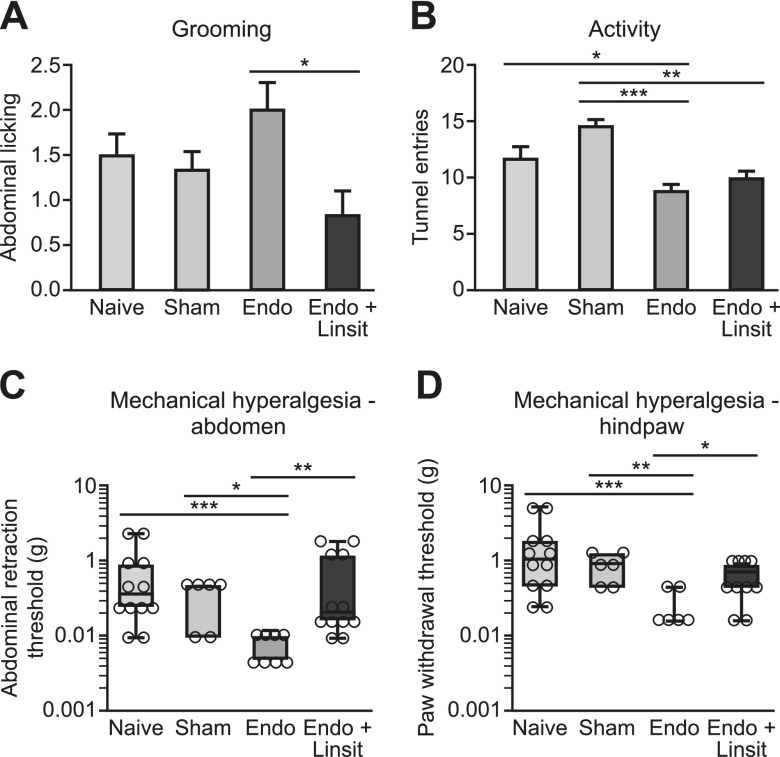
Igf-1r inhibition in mice with endometriosis rescues endometriosis-associated changes in sensory behavior. Endometriosis mice were treated with vehicle (*n* = 12) or 60 mg/kg linsitinib (*n* = 12) and compared with naive (*n* = 12) and sham-treated (*n* = 6) animals. *A*) In C57BL/6 mice with endometriosis, treatment with linsitinib significantly reduced spontaneous abdominal grooming compared with vehicle-treated endometriosis mice (*P* < 0.05). *B*) Exploratory behavior was significantly reduced in mice with endometriosis compared with naive (*P* < 0.05) and sham-treated controls (*P* < 0.001). Linsitinib treatment did not alter this. *C*, *D*) Mechanical withdrawal threshold shown by von Frey filament testing was measured on the abdomens (*C*) and plantar hind-paws (*D*) of mice with endometriosis. *C*, *D*) The withdrawal threshold (g) on both abdomen and hind-paw is decreased in mice with endometriosis compared with naive (*P* < 0.001) and sham-treated (*P* < 0.05, *P* < 0.01) animals. Linsitinib treatment significantly increased abdominal (*P* < 0.01) and hind-paw withdrawal thresholds (*P* < 0.05) compared with vehicle-treated mice. A single time point (d 24) that exhibited maximal changes in sensory behavior is shown. Statistical analysis was performed using a 1-way ANOVA and Tukey’s multiple comparison test (*A*, *B*) or a Kruskal-Wallis nonparametric test with Dunn’s multiple comparison test (*C*, *D*). Linsit, linsitinib. *P < 0.05, **P < 0.01, ***P < 0.001.

To further explore the role of Igf-1 in mice with endometriosis-associated pain, we inhibited Igf-1r using linsitinib, a selective IGF-1R inhibitor that prevents autophosphorylation and activation of downstream signaling. Treatment of endometriosis mice with 40 mg/kg linsitinib decreased grooming levels in mice with endometriosis (**Fig. 7*A***; *P* < 0.05). Although activity was reduced in mice with endometriosis, treatment with linsitinib did not rescue this behavioral change (Fig. 7*B*). Abdominal retraction (*P* < 0.01) and paw withdrawal thresholds (*P* < 0.05) were higher in endometriosis mice treated with linsitinib compared with vehicle-treated mice (Fig. 7*C, D*) such that there was no significant difference between linsitinib-treated and control animals. These findings indicate that inhibition of Igf-1 signaling in mice with endometriosis can attenuate endometriosis-associated-pain.

## DISCUSSION

Endometriosis is a common incurable disease with devastating impacts on health-related quality of life. Despite its prevalence, there remains only limited treatment options, and new therapeutic strategies are an area of unmet clinical need. Using a combination of *in vivo*, *ex-vivo*, and *in vitro* models, we show that macrophages play a key role in endometriosis-associated hyperalgesia, that disease-modified macrophages exhibit increased expression of IGF-1, and that inhibition of macrophage-derived IGF-1 can attenuate nerve growth and expression of nociceptive genes *in vitro*. Finally, we demonstrate that inhibition of Igf-1r in mice with endometriosis can attenuate hyperalgesia. Our study suggests that macrophages or signaling resulting from factors that they produce such as IGF-1 could be targeted to alleviate endometriosis-associated pain in women.

Pelvic pain is the most common presenting symptom of endometriosis (38). The pain is thought to be a manifestation of a 2-way dialogue between the nervous system and endometriosis lesions: the presence of endometrial-like tissue in the pelvic cavity of women with endometriosis causes inflammation (39), and lesions are innervated by sensory nerve fibers that sense this peripheral inflammatory environment and generate a nociceptive response resulting in pain perception. Macrophages are the most abundant immune cell present within the pelvic cavity (40), and in women with endometriosis, an increase in the number of macrophages both in the PF (41, 42) and in lesions (43) is evident; thus, these cells may contribute significantly to the inflammatory milieu present in the disease. In our study, we used an established mouse model of endometriosis to demonstrate that macrophage depletion using liposomal clodronate can attenuate endometriosis-associated spontaneous grooming and mechanical hyperalgesia (both locally and at a referred site). This finding complements and extends studies carried out in other rodent models of disease-related chronic pain that report a role for macrophages and monocytes in the generation of pain-related behaviors (19−21).

We have previously established that the presence of endometriosis lesions in mice causes molecular changes in the CNS (14). In this study, we demonstrated that Cox-2 and Tnf-α were significantly elevated in the spinal cords and brains of mice with endometriosis and that depletion of peripheral macrophages attenuated expression of these inflammatory markers. The expression of proinflammatory cytokines and Cox-2 in the CNS is a biomarker of spinal neuroinflammation and centralized inflammatory pain (12, 35, 44, 45). Our data suggest that macrophages present in the pelvic cavity and in endometriosis lesions play a key role in establishing maladaptive changes in the CNS that are associated with enhanced pain perception. It was surprising that these changes could be reversed, suggesting that in our model and at the time-points analyzed, CNS alterations are dynamic. The extent of spinal neuroinflammation in endometriosis remains unknown, although recently it was shown that in a minimally invasive model, mice with endometriosis exhibit astrocyte activation and subtle changes in immunoreactivity of microglia (46). Further characterizations of spinal maladaptation’s characteristic of neuroinflammation in endometriosis are warranted.

Macrophages play diverse roles in health and disease in all tissues of the body and consequently exhibit tissue- and disease-specific transcriptional profiles (47). In line with this, peritoneal macrophages from women with endometriosis exhibit an enhanced activation state characterized by enhanced expression of pro- and anti-inflammatory cytokines (48). Studies in mice have demonstrated that lesion-resident macrophages exhibit a phenotype that promotes lesion growth and enhances vascularization of lesions (22, 23), suggesting that EAMs exist as a disease-modified population that exhibits a wound-healing phenotype. To investigate factors produced by macrophages that may be implicated in contributing to endometriosis-associated pain, we analyzed mRNA concentrations of neurotrophins in *in vitro* generated EAMs. We identified that IGF-1 was significantly up-regulated in EAMs compared with control macrophages. Of note, expression of IGF-1 is a key characteristic of macrophages exhibiting a tissue-repair or wound-healing phenotype (49). This supports the historical hypothesis that EAMs are prorepair (23). We confirmed expression of Igf-1 in lesion-resident macrophages in women with endometriosis and mice with induced endometriosis, and we hypothesized that macrophage-derived Igf-1 is likely to play a key role in the pathogenesis of the disease.

PF concentrations of IGF-1 have previously been shown to be elevated in patients with endometriosis compared with those without (50), and we confirmed this in the current study. IGF-1 present in the PF is thought to play a role in the pathophysiology of endometriosis by stimulating the growth of and preventing apoptosis of endometrial-like cells (51). In endometriotic stromal cells, IGF-1 up-regulates expression of estrogen receptor β and aromatase, important drivers of endometriotic pathogenesis (52). Although these limited studies have inferred a role for IGF-1 in the pathophysiology of endometriosis, the current study is the first to implicate a role for IGF-1 in endometriosis-associated pain. In support of our findings, we identified a positive correlation between PF IGF-1 concentrations and self-reported pain score in women with chronic pain with and without endometriosis, although there was no correlation with disease stage. There is increasing evidence that Igf-1 contributes to pain hypersensitivity through binding to Igf-1r and activating Igf-1r–mediated PI3K, ERK, protein kinase B, or MAPK intracellular signaling pathways (53). Igf-1r is widely expressed in small, medium, and large DRG neurons (37, 54), and in chronic inflammatory and tissue injury models, Igf-1 signaling enhances thermal and mechanical hyperalgesia (54, 55). Moreover, inhibition of Igf-1r can reverse mechanical allodynia and thermal hyperalgesia in a rat model of cancer bone pain (36). In the current study, Igf-1r inhibition had a profound effect on endometriosis-associated hyperalgesia by reversing endometriosis-associated spontaneous grooming as well as abdominal and hind-paw hyperalgesia, thus validating a strong association between IGF-1 signaling and endometriosis-associated pain.

Finally, we were able to infer a strong link between macrophage-derived IGF-1 and the observed endometriosis-associated changes in sensory behavior using mechanistic *in vitro* models. We demonstrated that EAMs enhance sprouting neurogenesis in rat DRG explants as well as promote increased expression of nociceptive genes in human stem cell–derived sensory neurons. IGF-1R inhibition attenuates these observed changes. Critically, enhanced expression of nociceptive genes is one of the first steps in generating primary afferent sensitization (10). We therefore suggest that in endometriosis lesions macrophage-derived IGF-1 contributes to pain by promoting nerve growth in lesions and by sensitizing nerves by enhancing nociceptive gene expression.

Global inhibition of IGF-1 or IGF-1R is likely to have many off-target effects due to the pleiotropic roles of IGF-1. Thus, we suggest targeting disease-promoting macrophages as a potential future treatment for endometriosis. We now know that macrophages play a key role in growth, vascularization, and innervation of endometriosis lesions as well as generating endometriosis-associated pain, placing these cells at the center of the pathophysiology of a complex disorder. There are many pre- and early clinical trials in cancer that are successfully testing immunotherapy, and key areas to target macrophages are *via* inhibition of recruitment, direct killing, or re-education of disease-modified macrophages (56). However, before this can be a possibility, it is vital for us to know more about EAMs, their heterogeneity, and how they differ from healthy macrophages required for normal physiologic processes.

In summary, our study supports a previously unrecognized critical role for macrophages in endometriosis-associated hyperalgesia. The data suggest that macrophage-derived IGF-1 is a key driver of hyperalgesia in the disorder by promoting neurogenesis and nerve sensitization. By targeting specific populations of macrophages that overexpress Igf-1, we may able to develop innovative new treatments for the debilitating pain associated with this common, incurable disease.

## Supplementary Material

This article includes supplemental data. Please visit *http://www.fasebj.org* to obtain this information.

## Abbreviations

Bdnf: brain-derived neurotrophic factor
Cy: cyanine
DRG: dorsal root ganglion
EAM: endometriosis-associated macrophage
EPHect: Endometriosis Phenome and Biobanking Harmonisation Project
hESC: human embryonic stem cell
IGF-1R: IGF-1 receptor
LpM: large peritoneal macrophage
Ly6C: lymphocyte antigen 6 complex, locus C
M0: unactivated macrophage
MDM: monocyte-derived macrophage
NGF: nerve growth factor
Nt-3: neurotrophin-3
PF: peritoneal fluid
PPP: picropodophyllin
qPCR: quantitative PCR
SCN: sodium voltage-gated channel
SpM: small peritoneal macrophage
TAC1: tachykinin precursor 1
TRP: transient receptor potential cation channel
