# Supplementary figures and images for: Macrophage-derived insulin-like growth factor-1 is a key neurotrophic and nerve-sensitizing factor in pain associated with endometriosis
Source: FASEB J. 2019 Jul 10;33(10):11210–22. doi: 10.1096/fj.201900797R (PMC6766660; doi:10.1096/fj.201900797R)

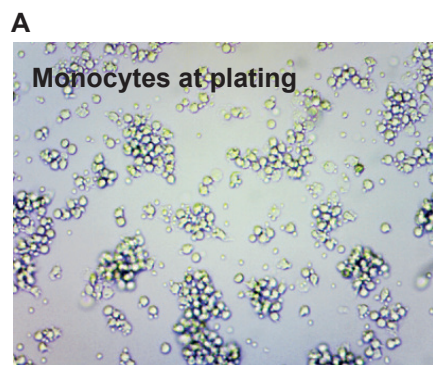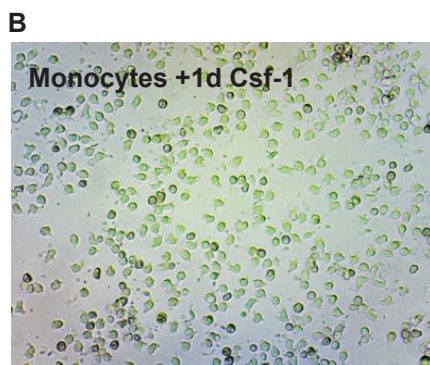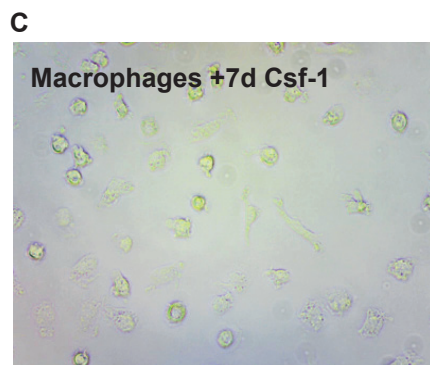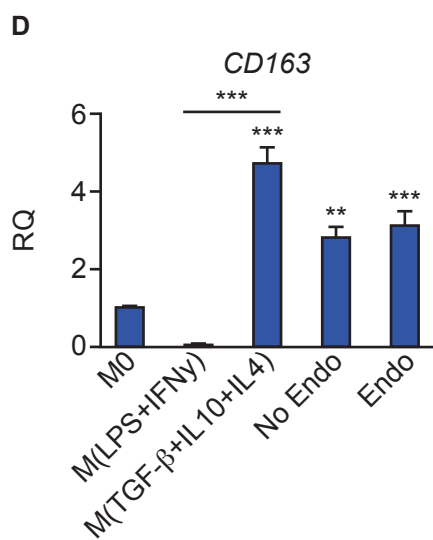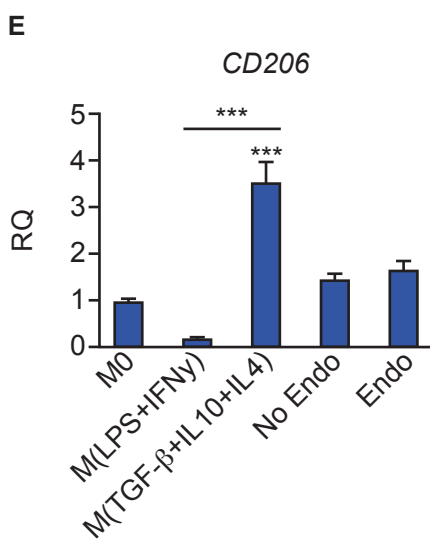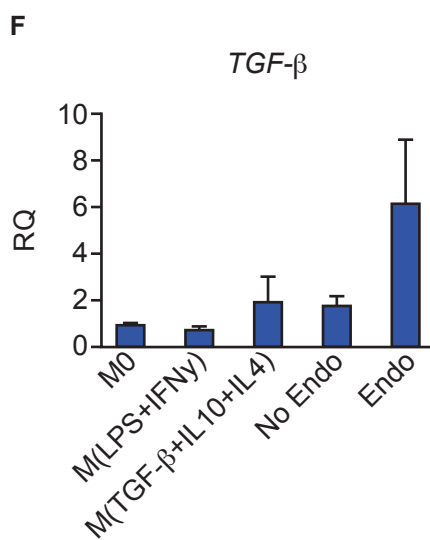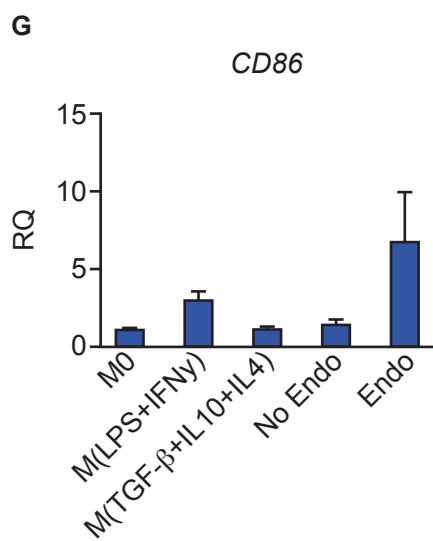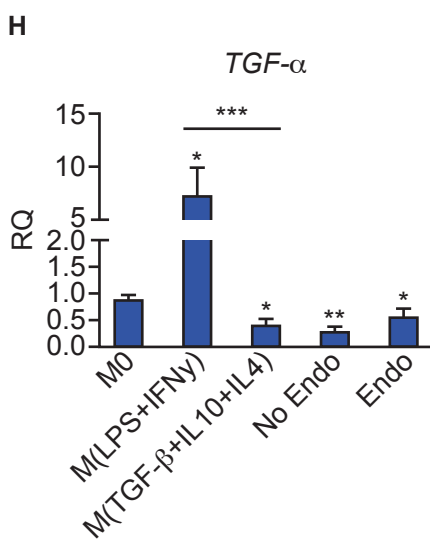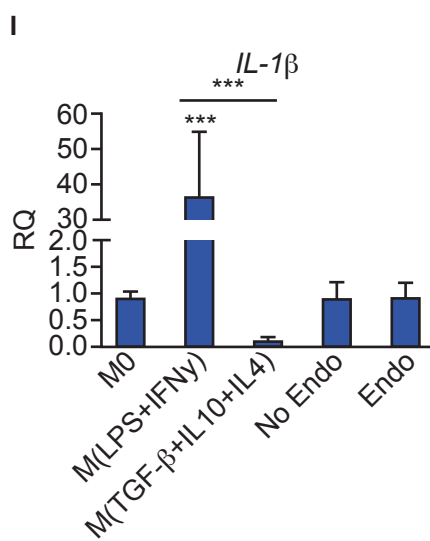

Supplement: Supplementary file 1 [file fj.201900797R.sf1.pdf]

A

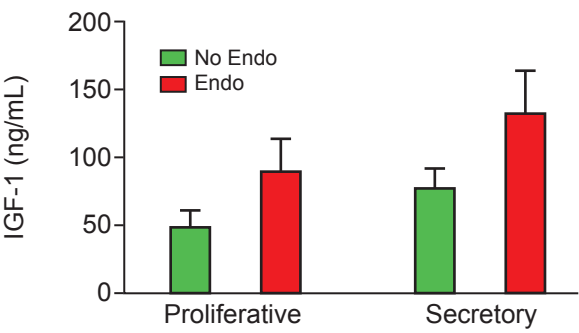

Supplement: Supplementary file 2 [file fj.201900797R.sf2.pdf]

**A***SCN3A*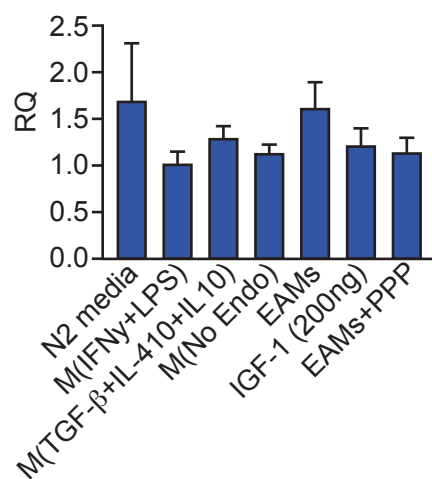**B***TRPV1*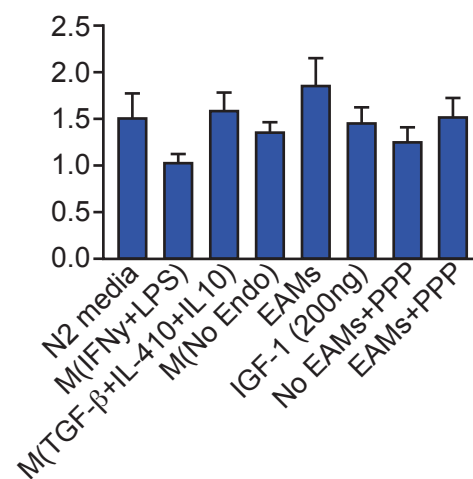**C***P2RX3*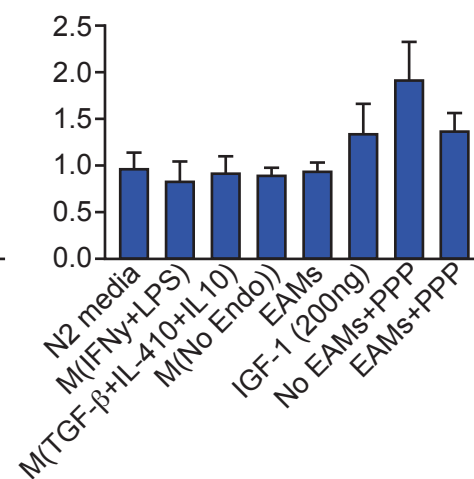**D***CGRP*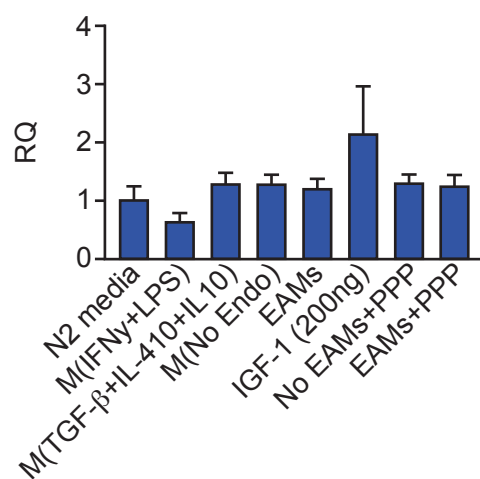

Supplement: Supplementary file 3 [file fj.201900797R.sf3.pdf]
