# Supplementary material for: Macrophage-derived insulin-like growth factor-1 is a key neurotrophic and nerve-sensitizing factor in pain associated with endometriosis
Source: FASEB J. 2019 Jul 10;33(10):11210–22. doi: 10.1096/fj.201900797R (PMC6766660; doi:10.1096/fj.201900797R)
Supplement: Supplementary file 4 [file fj.201900797R.sd1.docx]

**Supplementary figure legends**

**Fig.S1. Characterization of peripheral blood monocyte to macrophage differentiation. a-c)** Representative images of monocytes and macrophages at different stages of the differentiation protocol (n=7 healthy female volunteers). **a)** Isolated monocytes immediately after plating. **b)** Adherent monocytes 1 day after plating and the addition of recombinant Csf-1. **c)** Adherent macrophages 7 days after plating and incubation with Csf-1. Images captured though a X5 objective. Differentiated macrophages have a granulated appearance and some exhibit changes in morphology, displaying a spindle shape. **d-i)** QPCR was used to determine mRNA concentrations of different cell surface markers and cytokines in differentiated and activated macrophages. Following differentiation, macrophages were activated with cytokines such that they represented prototypical activations states; LPS+IFNγ to generate pro-inflammatory macrophages, TGFβ, Il-4, Il-10 to generate pro-repair macrophages. Some macrophages were also activated peritoneal fluid from women with (Endo, n=7) or without (no Endo, n=8) endometriosis. **d)** mRNA concentrations of *CD163,* **e)** *CD206* and **f)** *TGFβ* which are established markers of pro-repair macrophages and **g)** *CD86*, **h)** *TNFα* and **i)** *IL-1β* which are established markers of pro-inflammatory macrophages. For simplicity statistical significance has only been indicated for comparisons between un-activated macrophages (M0) and all other treatments (asterisks above bars), and comparisons between M(LPS+IFNγ) and M(TGF-β+IL-4+IL-10) or M(No Endo) and M(Endo), shown with horizontal bars and asterisks.

**Fig.S2. IGF-1 concentrations in peritoneal fluid of women with and without endometriosis. a)** ELISA analysis of peritoneal fluid from patients with endometriosis (n=13; ENDO, n= 6 proliferative phase and n=7 secretory phase) compared to patients without (n=9; NO ENDO, n=5 proliferative phase and n=4 secretory phase).

**Fig.S3. Expression of nociceptive genes in hESC derived human sensory neurons in response to incubation with conditioned media from EAMs.** Human sensory neurons were differentiated from H9 human embryonic stem cells using small molecular inhibitors as previously described^29^. QPCR analysis revealed that incubation of sensory neurons with ivEAM conditioned media did not later mRNA concentrations of **(a)** the sodium gated ion channel *SC39A,* **(b)** the vanilloid channel *TRPV1*, **(c)** the purinergic channel *P2RX3*or the neuropeptide *CGRP.*
